# Monte Carlo calculation based on hydrogen composition of the tissue for MV photon radiotherapy

**DOI:** 10.1120/jacmp.v16i5.5586

**Published:** 2015-09-08

**Authors:** Benjamin Demol, Romain Viard, Nick Reynaert

**Affiliations:** ^1^ Department of Radiotherapy Centre Oscar Lambret Lille France; ^2^ AQUILAB SAS Loos Les Lille France; ^3^ IEMN UMR CNRS 8520 Villeneuve d'Ascq France

**Keywords:** Monte Carlo, hydrogen content, stoichiometric calibration, megavoltage photon radiotherapy, magnetic resonance imaging

## Abstract

The purpose of this study was to demonstrate that Monte Carlo treatment planning systems require tissue characterization (density and composition) as a function of CT number. A discrete set of tissue classes with a specific composition is introduced. In the current work we demonstrate that, for megavoltage photon radiotherapy, only the hydrogen content of the different tissues is of interest. This conclusion might have an impact on MRI‐based dose calculations and on MVCT calibration using tissue substitutes. A stoichiometric calibration was performed, grouping tissues with similar atomic composition into 15 dosimetrically equivalent subsets. To demonstrate the importance of hydrogen, a new scheme was derived, with correct hydrogen content, complemented by oxygen (all elements differing from hydrogen are replaced by oxygen). Mass attenuation coefficients and mass stopping powers for this scheme were calculated and compared to the original scheme. Twenty‐five CyberKnife treatment plans were recalculated by an in‐house developed Monte Carlo system using tissue density and hydrogen content derived from the CT images. The results were compared to Monte Carlo simulations using the original stoichiometric calibration. Between 300 keV and 3 MeV, the relative difference of mass attenuation coefficients is under 1% within all subsets. Between 10 keV and 20 MeV, the relative difference of mass stopping powers goes up to 5% in hard bone and remains below 2% for all other tissue subsets. Dose‐volume histograms (DVHs) of the treatment plans present no visual difference between the two schemes. Relative differences of dose indexes $D_{98},D_{95},D_{50},D_{05},D_{02}$, and $D_{\text{mean}}$ were analyzed and a distribution centered around zero and of standard deviation below 2% (3σ) was established. On the other hand, once the hydrogen content is slightly modified, important dose differences are obtained. Monte Carlo dose planning in the field of megavoltage photon radiotherapy is fully achievable using only hydrogen content of tissues, a conclusion that might impact MRI dose calculation, but can also help selecting the optimal tissue substitutes when calibrating MVCT devices.

PACS numbers: 87.55.D‐, 87.55.dk, 87.55.K‐, 87.57.Q‐

## I. INTRODUCTION

In Monte Carlo treatment planning systems, tissue composition represented by density and elemental composition is required as a function of computed tomography (CT) numbers. Some current common practices to establish tissue characterization can introduce systematic errors in dose planning. Although density is often interpolated in a continuous way, the CT number scale is always divided into a finite number of subsets to link CT number to chemical elemental composition.^1^, ^2^ Usually, six or fewer media of average composition are defined (e.g., air, lung, fat, water, muscle and bone). In this way, some media are averaged on a large range of CT numbers (tissue compositions) (e.g., between soft bone and high density cortical bone the composition changes dramatically).^(3)^ It has been shown by Verhaegen and Devic^(4)^ that large dose errors (up to 10%) for MV photon beams can occur from inaccurate assignment of media. Moreover, tissue‐equivalent substitutes are often used to calibrate CT scanners just by matching their density to the density of real tissues. As their elemental composition diverges from that of real tissues, calibration curves of CT number to density can differ from reality.

In their work, Vanderstraeten et al.^(5)^ intended to deal with these two pitfalls using a stoichiometric CT calibration method, previously described by Schneider et al.^(6)^ This resulted in ten different bone subsets that were selected by dosimetric properties. From this work, a general inverse proportionality between hydrogen content and bone density of the obtained tissue composition subsets can be deduced. Furthermore, using the database of the National Institute of Standards and Technology (NIST) XCOM^(7)^ and ESTAR,^(8)^ one can easily notice that hydrogen differs from other human body elements in both mass attenuation coefficient and total mass stopping power. Mass attenuation coefficients for a mixture can be determined by the weighted sum of the constituent elements.^(9)^ Also, for the mass stopping powers of compounds, this method can be used as a close approximation.^(10)^ As these two quantities govern dose deposition in a Monte Carlo algorithm, it is reasonable to assume that only the hydrogen content could be sufficient for dose calculation in human tissues, and it is considered as the main hypothesis of this study.

In the current work, we demonstrate that, for megavoltage photon radiotherapy, only the hydrogen content of the different tissues is of interest. Treatment plans on the CyberKnife accelerator have been used in this study to estimate the accuracy of such a hypothesis. Current observations might have an impact on MRI‐based dose calculations and on MVCT calibration using tissue substitutes.

## II. MATERIALS AND METHODS

### A. Stoichiometric model: photon attenuation and energy deposition characteristics of dosimetric tissue subsets

The cornerstone of our work is based on the stoichiometric calibration method described by Vanderstraeten et al.^(5)^ and Schneider et al.,^(6)^ and applied to our Aquilion 16LB CT scanner (Toshiba Medical Systems, Otawara, Japan). The aim of this method is to divide tissue materials into more dosimetric subsets than usually described, in order to depict tissue diversity in a better way. The first step of this method is to determine the empirical parameters of the total attenuation coefficient (in the CT beam) from the contribution of each individual chemical element within the following system of equations:

(1) $$\mu =\rho N_{A}\sum_{i=1}^{n}\left(\frac{w_{i}}{A_{i}}(K^{ph}Z_{i}^{4.62}+K^{coh}Z_{i}^{2.86}+K^{KN}Z_{i})\right)$$

(2) $$H=1000\left(\frac{\mu }{\mu_{water}}-1\right)$$

with *ρ* the mass density, $N_{A}$ the Avogadro constant, $w_{i}$ the elemental weight of the element $i,A_{i}$ its atomic mass, $Z_{i}$ its atomic number, and *H* the CT number; $K_{ph},K_{coh}$, and $K_{KN}$ are the parameters to be determined experimentally for our CT beam (they are CT scanner‐dependant). They characterize respectively the abundance of the photoelectric effect, of the Rayleigh scattering, and of the Compton scattering, in terms of cross section.

For this step, the so‐called cheese phantom (Accuray Inc., Sunnyvale, CA) with 13 inserts of known density and elemental composition was scanned (120 kVp beam, field of view of $70\;\text{cm},1.0\times 1.37\times 1.37\;{\text{mm}}^{3}$ voxel size) and the corresponding CT numbers were measured. The parameters were determined by a multiple linear regression and they are given here for information: $K^{\text{PH}}/K^{\text{KN}}=3.51\times 10^{-5};K^{\text{coh}}/K^{\text{KN}}=-1.82\times 10^{-3}$. Once the parameters are defined, the (1), (2) can be used to calculate the CT number of any tissue of known density and elemental composition. A table of more of 70 tissues classified by Woodard and White^(3)^ was first used in this way to establish the mass density calibration curve.

**Figure 1:**
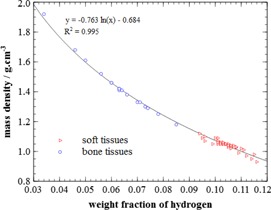
Correlation between hydrogen content and mass density of the human tissues provided by Woodard and White.^(3)^

The second step of the stoichiometric method in Vanderstraeten et al.^(5)^ consists of gathering these tissues into discrete dosimetric tissue subsets with similar properties in function of CT number. Composition of theses subsets is obtained by the mean composition of the tissues in the subset. A general relation between hydrogen content and density is observable (Fig. 1). The hydrogen weight fraction of a subset is close to that of the tissues composing this subset (small standard deviation). The number and the range of the subsets in term of CT number depend on a dosimetric criterion which states that the Monte Carlo simulated depth dose ratios in a large cubic homogenous phantom from two adjacent subsets (with mass density set to $1\;g.{\text{cm}}^{-3}$) do not differ by more than 1%. In the current work, dosimetrically equivalent tissue subsets are generated using Monte Carlo calculations in homogeneous phantoms and from preconceived results of Vanderstraeten et al.^(5)^ regarding the approximate number of intervals and the boundaries between them. Simulations were performed with DOSXYZnrc^(11)^ using a phase space of a CyberKnife (Accuray Inc., Sunnyvale, CA) unit, previously modeled.^(12)^ The result is an almost continuously changing tissue composition in consecutive subsets and, thus, a continuously changing hydrogen content, with a continuously changing density (from the mass density calibration curve). This specific calibration scheme will be considered as the reference scheme.

To demonstrate the importance of hydrogen, a dosimetric subset scheme based on the original was generated by keeping the correct hydrogen weight fraction in all subsets and by allocating the remaining weight fraction to oxygen (replacing all nonhydrogen elements by oxygen). Only the hydrogen content is considered this way. This scheme will be denoted the HO (Hydrogen Oxygen) scheme.

Alternatively, a third scheme was derived from the original by keeping a constant hydrogen weight fraction of 11% within each subset. The difference between the actual hydrogen weight and current value is transferred to the oxygen fraction. This scheme was named the HA (Hydrogen Adipose) scheme, as adipose has a hydrogen weight fraction of 11%. In this scheme all nonhydrogen elements are modeled correctly (except oxygen). This scheme is expected to provide noticeable deviations from the reference scheme under the assumption that the hydrogen content mostly governs particle interactions.

The mass attenuation coefficients and total mass stopping power have been calculated using the NIST databases and have been compared for the three schemes in order to provide primary indications of the validity of the main hypothesis.

### B. Monte Carlo dose calculations

An in‐house developed Monte Carlo system based on MCDE^(13)^, called MODERATO, is currently used in our center as a dose verification tool for quality assurance for all CyberKnife treatment plans performed in our radiotherapy department. This system is based on the EGSnrc^(14)^ code and its simulation engines BEAMnrc^(15)^ for the accelerator phase space, and DOSXYZnrc^(11)^ for the particle transport in the patient geometry. This required a professional reprogramming of MCDE to obtain a more robust user‐friendly system using a JavaScript GUI that allows launching the calculations on different servers, and evaluating the obtained results. Different Monte Carlo engines (EGS, Geant4^(16)^) can be selected for different parts of the simulation (e.g., Geant4 can be used to determine the phase space, and EGS to perform the dose calculation in the patient geometry, or vice versa).

The CyberKnife accelerator has been modeled using BEAMnrc (and the egs++ geometry package to model the iris collimator), generating phase space files at the exit plane of the secondary collimator.^(12)^ The system allows selecting a treatment plan established on the MultiPlan (Accuray, Inc.) treatment planning system (TPS), and to compare the dose distribution and dose‐volume histograms (DVH) between MultiPlan and the Monte Carlo dose engine.

The different dosimetric subset schemes were retrospectively applied to patient treatment plans using the Monte Carlo system. The simulations were realized on 25 CyberKnife treatments divided in anatomical locations, according to Table 1. Different anatomical areas were used to cover a large panel of tumor tissues and tissues near the tumor as, for example, the bone metastasis, which allow testing the modelization of the bone subsets for the HO scheme.

CyberKnife treatment plans are composed of more than 100 beams with diameters between 7.5 mm and 60 mm. CTs were scanned at a resolution of 1×1×1 mm3 and the resolution of the dose voxels in the Monte Carlo system was set at 2×2×2 mm3. The number of histories simulated was fixed to $10^{9}$ to get a statistical uncertainty below 1% within at least 95% of the voxels in the planning target volume (PTV). Dose calculations using the Monte Carlo system were performed using the three dosimetric subset schemes for each treatment plan.

Comparison analyses of dose indexes from the DVH were performed. It was considered as the optimal tool to compare dose results since optimization and evaluation of treatment plans are routinely based on DVHs. DVH, dose repartition, and dose profiles along the PTV are presented for one CyberKnife treatment plan, allowing a more detailed analysis.

**Table 1:** Anatomical repartition of the treatment plans. PTVs in the head are localized in soft tissues or close to bone of the skull. The pelvis group gathers prostate, cervix, endometrium, and anus. The bone metastasis group gathers vertebra, femoral head, pelvic bone, and femur

*Localization*	*CyberKnife*
lung	6
head	10
pelvis	2
pancreas	1
ENT (ear, nose, throat)	1
bone metastasis	5
Total	25

## III. RESULTS

### A. Stoichiometric model: photon attenuation and energy deposition characteristics of dosimetric tissue subsets

**Figure 2:**
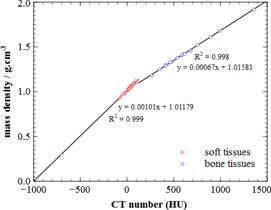
Mass density calibration curve. The equations of linear regression are displayed on the graph. $R^{2}$ is the correlation coefficient. Tissue CT numbers are calculated using the stoichiometric model.

Discrepancies between the CT numbers calculated from the stoichiometric model and the measured values are presented in Table 2, allowing a qualitative appreciation of the coherence between the model and the measures. There are globally no significant discrepancies and the results are in better agreement than in Vanderstraeten et al.^(5)^ The density calibration is presented in Fig. 2. The points represent the CT number of the tissues calculated using the stoichiometric model and the Woodard and White tables.^(3)^ The correlation coefficients confirm the validity of the linear fit. As also the case for the more conventional calibration model (using tissue substitutes) the curve is not continuous at 100 HU, but the difference of mass density which could occur from a misattribution of tissue around this point is only $0.03\;g.{\text{cm}}^{-3}$, which can be considered negligible.

The application of the second part of the method of Vanderstraeten et al.^(5)^ led to the dosimetric tissue subsets shown in Table 3 constituting the reference scheme, which are consistent with their results. The weight fraction of hydrogen and the density within subsets agree with the previous observation of Fig. 1 that there is an inverse proportionality between these quantities, except for the lung subset which is particular in its definition. Lung tissue in the Woodard and White table has a density of 1.05 and a weight fraction of hydrogen of 10.3%, comparable to muscle tissue, for example. To simulate the partial volume effect of the voxelization of lung with air due to the small structure of pulmonary alveolus and pulmonary bronchus compared to the voxel size, density is artificially lowered within the lung subset without changing the composition. Because of that, the inverse proportionality is not highlighted within Table 3 although it actually exists.

**Table 2:** Differences in terms of CT numbers between measures ($H_{\text{measured}}$) and calculations ($H_{\text{calculated}}$) using the stoichiometric calibration of the inserts of the cheese phantom. CT numbers are expressed in HU

*Insert Denomination*	*Mass Density* ($g.{\text{cm}}^{-3}$)			*Residual Error*
		$H_{measured}$	$H_{calculated}$	$H_{measured}-H_{calculated}$
LN300	0.28	−713	−713	14
LN450	0.48	−713	−713	−713
adipose	0.945	−713	−713	−713
breast	0.984	−713	−713	−713
solid water	1.019	−713	7	−713
brain	1.051	13	35	−713
liver	1.094	76	90	−713
inner bone	1.15	223	196	27
B‐200 bone	1.157	222	200	22
CB2‐30%	1.335	476	461	15
CB2‐50%	1.561	856	835	21
cortical bone	1.824	1253	1273	−713

To illustrate the generation of the two additional designed schemes from the reference scheme, the construction of the bone6 subset of the HO scheme and the HA scheme is given in Table 4 as an example. In this subset, the error induced on the weight fraction of hydrogen in the HA scheme is almost twice compared to the actual value of 5.6% in the HO scheme.

**Figure 3:**
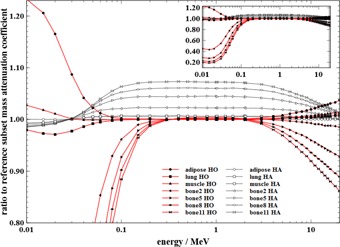
Ratio to reference subset mass attenuation coefficients of some subsets for the HO scheme and the HA scheme. Not all subset curves have been displayed, to improve clarity. The top right window shows the same curves on a higher vertical range.

The following representations of the mass attenuation coefficients and the mass stopping powers of both the HO and the HA schemes were normalized to those of the reference stoichiometric calibration scheme, allowing better visualization of the impact of the applied modifications in tissue composition. The mass attenuation coefficients of several subsets of the HO and HA schemes are represented in Fig. 3. Not all subsets have been displayed, for clarity. The hidden bone curves follow the same trend as displayed. Three main parts can be distinguished for both the HO and the HA schemes.

The most important part is the energy range between 200 keV and 3 MeV, where the reference scheme and the HO scheme present almost the same attenuation throughout all subsets. Maximum difference in this range is about 1% for bone11 — namely the subset with the lowest hydrogen content. This result is consistent with the results obtained by Seco and Evans^(17)^ who have demonstrated that, in this energy range, the attenuation is proportional to electron density. Electron density is proportional to the weighted ratio of atomic number to atomic weight $Z_{i}/A_{i}$, which roughly equals 0.5 for all chemical elements of the human body, except for hydrogen having a ratio of 1. Replacing elements (except hydrogen) by oxygen does not modify the weighted ratio of $Z_{i}/A_{i}$ nor the attenuation.

**Table 3:** Elemental composition of dosimetric tissue subsets of the reference scheme. Subsets are represented by a name, a CT number lower limit $H_{l}$, a CT number upper limit $H_{u}$, an atomic weight fraction $w_{i}$, and a continuous mass density calculated from the mass density calibration curve. The density provided in the last column is the mean density of the tissues within the subset and is given to show the inverse proportionality between density and hydrogen content

*Subset Name*	$H_{l}$	$H_{u}$	$w_{H}$	$w_{C}$	$w_{N}$	$w_{O}$	$w_{Na}$	$w_{Mg}$	$w_{P}$	$w_{S}$	$w_{Cl}$	$w_{K}$	$w_{Ca}$	*Density*
lung	−713	−713	0.103	0.101	0.029	0.755	0.002	0	0.002	0.003	0.003	0.002	0	0.299
adipose	−713	20	0.11	0.345	0.015	0.523	0.002	0	0.001	0.001	0.002	0.001	0	1.001
muscle	20	80	0.103	0.143	0.031	0.713	0.002	0	0.002	0.002	0.002	0.002	0	1.054
bone1	80	200	0.095	0.153	0.042	0.683	0.0055	0	0.011	0.0075	0.003	0	0	1.105
bone2	200	320	0.085	0.405	0.028	0.367	0.001	0.001	0.034	0.002	0.002	0.001	0.074	1.192
bone3	320	440	0.075	0.294	0.037	0.45	0.001	0.001	0.044	0.002	0.001	0.001	0.094	1.278
bone4	440	560	0.069	0.264	0.037	0.46	0.001	0.001	0.053	0.001	0.001	0.001	0.112	1.343
bone5	560	680	0.062	0.291	0.035	0.402	0.001	0.001	0.065	0.002	0.001	0	0.14	1.433
bone6	680	800	0.056	0.235	0.04	0.434	0.001	0.001	0.072	0.003	0.001	0.001	0.156	1.485
bone7	800	920	0.05	0.212	0.04	0.435	0.001	0.002	0.081	0.003	0	0	0.176	1.610
bone8	920	1040	0.046	0.199	0.041	0.435	0.001	0.002	0.086	0.003	0	0	0.187	1.677
bone9	1040	1160	0.042	0.184	0.041	0.435	0.001	0.002	0.092	0.003	0	0	0.2	1.748
bone10	1160	1300	0.038	0.169	0.041	0.435	0.001	0.002	0.098	0.003	0	0	0.213	1.835
bone11	1300		0.034	0.155	0.042	0.435	0.001	0.002	0.103	0.003	0	0	0.225	1.919

**Table 4:** Composition of the bone6 subset for the three schemes under consideration

*Scheme*	$w_{H}$	$w_{C}$	$w_{N}$	$w_{O}$	$w_{Na}$	$w_{Mg}$	$w_{P}$	$w_{S}$	$w_{Cl}$	$w_{K}$	$w_{Ca}$
Reference	0.056	0.235	0.04	0.434	0.001	0.001	0.072	0.003	0.001	0.001	0.156
HO	0.056			0.944							
HA	0.11	0.291	0.035	0.380	0.001	0.001	0.065	0.002	0.001	0	0.14

Deviations arise above 3 MeV within all subsets, with larger deviations for bone tissues, as shown by the example of bone11 with a ratio of 0.86 at 20 MeV. Seco and Evans^(17)^ have also shown pair production becomes important above 10 MeV, especially for high Z elements such as calcium. A certain part of the pair production is lost when calcium is not considered within the HO scheme and leads to a decrease of attenuation. Nevertheless, the use of the ratio virtually intensifies these discrepancies above 10 MeV; in this range, discrepancies ranging from 5% to 15% between mass attenuation coefficients of bone can be noticed. As the curve of the mass attenuation coefficient has a negative slope for these subsets and is on the order of $10^{2}$ beyond 10 MeV, the relative difference, for instance, between bone11 (reference scheme) and bone11 (HO scheme) of absolute attenuation, is less than 2.5% for a distance of 5 cm. Such differences are difficult to translate to dosimetric impact, but they seem negligible. Furthermore the energy spectrum of the CyberKnife^(18)^ has a small part of photons between 3 MeV and about 6 MeV.

Deviations also occur below 300 keV within all subsets, especially for the mass attenuation coefficient of bone tissues, whose ratio is a factor 5 at 20 keV. Calcium content within the stoichiometric scheme is the origin of such deviations. Indeed the high atomic number of calcium provides more attenuation by the photoelectric effect in this energy range. Although the energy spectrum of the CyberKnife is centered around 1 MeV and does not contain a lot of photons of these energies,^(18)^ a proportion of photons could reach such energies from scattering or Bremsstrahlung. This could cause some deviations in the dose calculation, but the effects of these discrepancies on the dose calculation are difficult to predict and may be weak.

Contrarily, even if the HA scheme curves are more faithful within the energy range extremities, they differ from the reference within the main energy range from 2.2% for the bone2 subset, 4.5% for the bone5 subset, 6.0% for the bone8 subset, and 7.3% for the bone11 subset at 1 MeV. These discrepancies remain almost constant between 100 keV and 5 MeV. The adipose subset ratio is unity along the energy range because the hydrogen weight fraction of the adipose subset is 11%. These discrepancies were expected as the hydrogen weight fraction difference with the reference scheme increases. Calcium content has only an effect for low energies and energies above 10 MeV as shown before, and has almost no impact on the mass attenuation coefficient and the mass stopping power between these thresholds. The HA scheme should introduce noticeable deviations from the stoichiometric scheme within bony targets.

**Figure 4:**
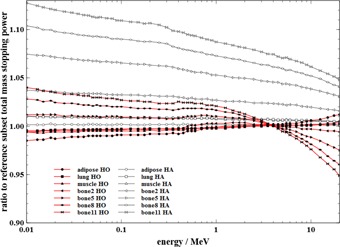
Ratio to reference unrestricted mass stopping powers of some subsets for the HO scheme and the HA scheme.

The total mass stopping powers for both the HO and HA schemes are displayed in Fig. 4. The mass stopping power curves show larger differences among the subsets and energies. The soft tissue subsets from both the HO and the HA schemes have approximately the same mass stopping power as the reference calibration scheme throughout the energy range; the deviations are well below 2%.

The bone2 subset from the HO scheme is consistent within 0.6% throughout the energy range. The bone5 subset from the HO scheme has a mass stopping power that differs by less than 1.3% from 10 keV to 12 MeV and reaches a 2.5% difference at 20 MeV. The difference in mass stopping powers within the bone8 subset from the HO scheme is between 3% and 2% from 10 keV to 100 keV, near 1.8% up to 1 MeV, progressively decreases to 0% at 4 MeV and to −4% at 20 MeV. The differences in mass stopping powers are 4% at 10 keV, 2% at 1 MeV, near 0% at 4 MeV, −1% at 7 MeV, and 5% at 20 MeV within the bone11 subset from the HO scheme. This spread of discrepancies in stopping powers is more difficult to analyze, and the prediction of the impact on the dose calculation is more challenging. The energy range from about 6 to 20 MeV should not be taken into account for the CyberKnife. The differences are, however, small for the energies of interest. These observations on the attenuation coefficient and the stopping power will lead to an overall result which will be studied in the following section.

For the HA scheme, the bone subset curves deviate as a function of the difference in hydrogen content. Even the soft bone subsets suffer from this difference — 2.7% difference at 1 MeV for the bone2 subset, for instance. For the bone11 subset, the discrepancy goes up to 8.7% at 1 MeV. These discrepancies in the HA scheme would lead most likely to erroneous results in bony structures and point out the importance of the hydrogen content.

### B. Monte Carlo dose calculations

**Figure 5:**
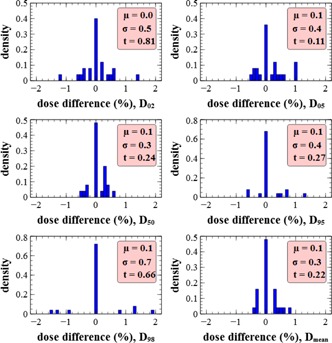
Density of relative dose difference of several dose indexes between the reference scheme and the HO scheme, for all treatments. Dose difference is expressed as $(D_{X,\text{ref}}-D_{X,\text{HO}})/D_{X,\text{ref}}$. Density is normalized to unity. μ is the mean of the distribution, σ is the standard deviation of the distribution, and t is the p‐value of the distribution for the null hypothesis (mean equal zero).

$D_{02},D_{05},D_{50},D_{95},D_{98}$, and $D_{\text{mean}}$ of the PTV were evaluated for each treatment plan using the three dosimetric tissue subset schemes. A distribution of the sample has been established for each dose index. The main hypothesis is that there is no difference between the HO scheme and the reference scheme (i.e., the null hypothesis corresponds to the mean of the population being zero). A threshold of 0.05 (p‐value) is used for the *t*‐test for significant divergence of the null hypothesis. Complementary the standard deviation of the population should stay below a defined threshold, defined as 3σ, should be inferior to 2% (to have 99% of the values below 2%). For simplicity, the standard deviation of the population has been estimated as the standard deviation of the sample. Relative difference of these indexes between the reference and the HO scheme are presented in Fig. 5. The red windows present the summary of the characteristics of the sample. The mean of the difference is comprised between 0% and 0.1%, which is consistent with the null hypothesis. All p‐values are above the defined threshold, which further validates the null hypothesis. The low spread of the differences for each dose index explains the low standard deviation. This complies with our defined criterion of 2% (3σ), except in $D_{98}$. However, a systematic error in small targets seems to be present in the dose index because of the small number of voxels inside the PTV. In spite of this eventual error, the value of 2.1% (3σ) has been accepted. The positive extreme value of the $D_{98}$ distribution (1.9%) arises from a bone metastasis case (vertebra) which is presented as an example in 6, 7. This small discrepancy can be attributed to a lack of calcium within the bone region when using the HO scheme. However this difference in $D_{98}$ is hardly visible in the DVH and is not clinically significant, as it will not influence the validation or the optimization of a treatment plan. The DVHs obtained for the HO and the reference scheme are hard to distinguish, indicating a good agreement. In general, all the DVHs compared present the same trend of quasi‐superposition between the two curves and are, therefore, clinically equivalent. There is a concordance between the two schemes, even in the bone regions and in regions close to the skin, and the behavior of attenuation at low energies did not introduce a significant bias. These overall results suggest that modeling of tissue by only density and hydrogen weight fraction is well suited for accurate dose calculations within any type of tissue for the CyberKnife treatments.

**Figure 6:**
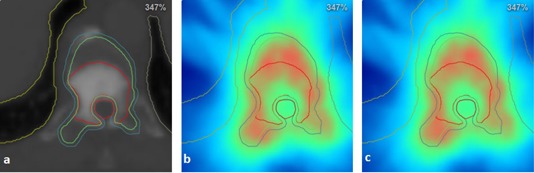
Delineation of the GTV (red contour), the CTV (green contour), and the PTV (blue contour) comprising the bone marrow (brown contour) of a T11 vertebra palliative treatment. Dose repartition (a), using the reference scheme (b) and using the HO scheme (c). Color scale goes to dark blue (0 Gy) to green (23 Gy–30 Gy) up to intense red (42 Gy). $D_{\text{mean}}$ to PTV: 32.1 Gy. Maximum dose to bone marrow is under 30 Gy.

Dose repartition can be evaluated qualitatively in Fig. 6 for the same vertebra case. Local higher dose (in intense red) are linked into the two repartitions, and there is no visual discrepancy. Dose profiles were drawn through the PTV and are presented in 8, 9. The profiles of the two schemes follow the same trend with some local discrepancies which are statistical. These local differences are, however, averaged on the entire PTV as the previous results indicated.

Relative difference of dose indexes between the reference and the HA scheme are presented in Fig. 10. The same comparisons were made as described above, although only a few results are needed to prove that the HA scheme is not equivalent to the reference scheme. For example, the p‐value of $D_{02},D_{05},D_{50}$, and ±1% in dose indexes are obtained within targets of soft tissue when there is no bone in close proximity. This was predictable and due to the intrinsic constitution of the HA scheme in terms of hydrogen content.

**Figure 7:**
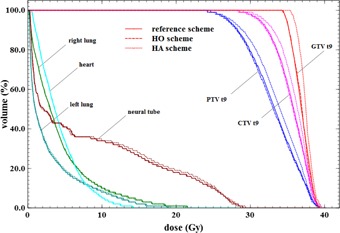
DVH of a vertebra target case. One color (online version) corresponds to one volume. Full line corresponds to the reference scheme, dashed to the HO scheme, dotted to the HA scheme.

**Figure 8:**
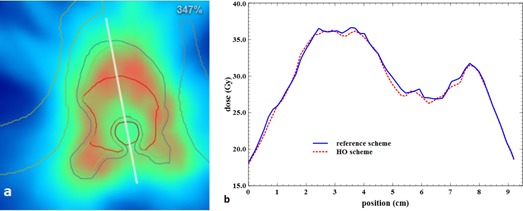
Line profile (top to bottom) crossing the tumor and the bone marrow of the same treatment plan as that of Fig. 6 (a), and dose value along this line for the reference scheme and the HO scheme (b).

**Figure 9:**
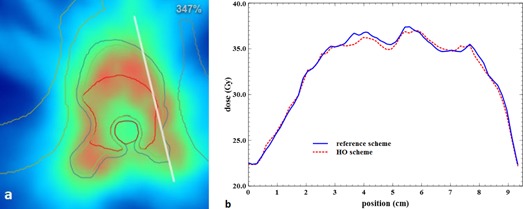
Line profile (top to bottom) crossing the tumor of the same treatment plan as that of Fig. 6 (a), and dose value along this line for the reference scheme and the HO scheme (b).

**Figure 10:**
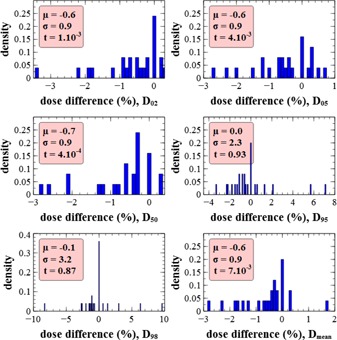
Density of relative dose difference of several dose indexes between the reference scheme and the HA scheme for all treatments. Dose difference is expressed as ($D_{X,\text{ref}}-D_{X,\text{HA}})/D_{X,\text{ref}}$. Density is normalized to unity. μ is the mean of the distribution, σ is the standard deviation of the distribution, and t is the p‐value of the distribution for the null hypothesis (mean equal zero).

## IV. DISCUSSION

The main hypothesis postulated after studying the attenuation coefficients and the stopping powers has been verified by Monte Carlo dose calculations using the HO scheme. There are small differences in dose indexes and DVHs between the stoichiometric scheme and the HO scheme even within the bone structures. The quasi‐superposition of the DVH curves for these two schemes in all studied cases is an important result, since the DVH is the main tool for evaluation and optimization of a treatment plan. These overall results prove that modeling of tissue by hydrogen weight fraction and density only is suitable for accurate dose calculations within any tissue type of the target for the CyberKnife treatments. As the CyberKnife beam is produced without any hardening filter, the energy spectrum contains a higher component of photons with energy around 0.6–1 MeV, compared to a conventional 6 MV spectrum. As mass attenuation coefficients and mass stopping powers for the HO scheme are in good agreement in this energy range (as seen in 3, 4), using a conventional 6 MV spectrum will not produce any alteration in the dose results. Previous conclusion can thus be applied more generally in the megavoltage photon radiotherapy field. However, no study on higher nominal energy like 18 MV or 20 MV was initiated due to the current trend to use these types of beams infrequently, particularly in the fields of IMRT and stereotaxy.

This result can be of prime interest in MRI‐only based Monte Carlo dose calculation, which is of great interest, taking into account the high tissue contrast of MRI for target and organ delineation and removing the uncertainty induced by the CT to MRI registration. The main obstacle hampering the usage of MRI as the unique modality in treatment planning is the lack of information about electron density. Current methods to derive electron density from MRI data by generating a pseudo‐CT are divided into two approaches: the anatomy‐based and the voxel‐based methods. In the anatomy‐based methods, deformable registration of reference paired MRI/CT scan (atlas or reference patient) to the MRI scan of the patient under consideration is used to build a pseudo‐CT from the warped reference CT scan^19^, ^20^, ^21^. Although mathematical or statistical operations could be used to smooth registration errors due to interpatient anatomical differences,^19^, ^20^ these methods fail for patients presenting high anatomy dissimilarities. The voxel‐based methods convert MRI intensity into pseudo‐CT number using either a bulk density assignment after segmenting tissues or using a regression model to compute continuous values of CT number from a set of MRI data.^22^, ^23^, ^24^, ^25^ Ultrashort echo time sequences^(26)^ are usually used in voxel based methods to distinguish the signal of bone from air. As the signal is acquired very close to the end of the excitation in UTE imaging, almost no supplemental weighting of the signal is added to the proton density weighting, and a link could be identified between UTE data and hydrogen density.^(27)^ This may open the way to a voxel‐based method to convert MRI data to hydrogen content, which could then be used for treatment planning by the method presented in this study. This method would rely on a physical basis whereas all the current methods rely on mathematical models. This work is, however, outside the scope of this study.

Another field of interest for this study is the use of tissue substitutes to calibrate MVCT devices for adaptive treatment planning. For adaptive tomotherapy, for example, MVCT is required to calculate dose on the patient geometry during treatment delivery. A calibration curve is needed to link MVCT intensities to tissue density. Phantom inserts that are considered tissue‐equivalent for kVCT calibration (determined by the high‐Z component) are not necessarily optimal for MVCT calibration. As demonstrated in the current paper, the degree of tissue equivalence is determined by the hydrogen content, while the high‐Z elements have no impact. However, sometimes discrepancies in hydrogen content can be compensated by changing the density as, for example, for Solid Water (RMI 457, Gammex, Middleton, WI). This issue has to be investigated in more detail.

## V. CONCLUSIONS

Our results are in agreement with our hypothesis that hydrogen weight fraction and density suffice to perform accurate Monte Carlo dose calculations within any tissue type in megavoltage photon radiotherapy conditions. The calcium content of bone tissues is not required for Monte Carlo simulation of a 6 MV beam. We have linked these results to mass attenuation coefficient and mass stopping power properties of designed subset schemes, which explains the success of this method. This outcome may be of high importance in the context of an MRI‐only radiotherapy workflow. Indeed, MRI is based on the concentration of protons inside tissues, so a method could be designed to extract hydrogen content from one or several MRI sequences. This result can also be used in the field of calibration of MVCT devices to select optimal tissue substitutes. This selection should be based on the hydrogen content instead of the high‐Z elements that are important for KVCT calibration.
